# Forecasting the publication and citation outcomes of COVID-19 preprints

**DOI:** 10.1098/rsos.220440

**Published:** 2022-09-28

**Authors:** Michael Gordon, Michael Bishop, Yiling Chen, Anna Dreber, Brandon Goldfedder, Felix Holzmeister, Magnus Johannesson, Yang Liu, Louisa Tran, Charles Twardy, Juntao Wang, Thomas Pfeiffer

**Affiliations:** ^1^ New Zealand Institute for Advanced Study, Massey University, Auckland, New Zealand; ^2^ Michael Bishop Consulting, Ottawa, Canada; ^3^ John A. Paulson School of Engineering and Applied Sciences, Harvard University, Cambridge, MA, USA; ^4^ Department of Economics, Stockholm School of Economics, Stockholm, Sweden; ^5^ Department of Economics, University of Innsbruck, Innsbruck, Austria; ^6^ Gold Brand Software, LLC, Herndon, VA, USA; ^7^ Department of Computer Science and Engineering, University of California, Santa Cruz, CA, USA; ^8^ Jacobs Engineering Group Inc., Herndon, VA, USA; ^9^ C41 & Cyber Center, George Mason University, Fairfax, VA, USA

**Keywords:** preprinting, forecasting, science policy

## Abstract

Many publications on COVID-19 were released on preprint servers such as *medRxiv* and *bioRxiv.* It is unknown how reliable these preprints are, and which ones will eventually be published in scientific journals. In this study, we use crowdsourced human forecasts to predict publication outcomes and future citation counts for a sample of 400 preprints with high *Altmetric* score. Most of these preprints were published within 1 year of upload on a preprint server (70%), with a considerable fraction (45%) appearing in a high-impact journal with a journal impact factor of at least 10. On average, the preprints received 162 citations within the first year. We found that forecasters can predict if preprints will be published after 1 year and if the publishing journal has high impact. Forecasts are also informative with respect to *Google Scholar* citations within 1 year of upload on a preprint server. For both types of assessment, we found statistically significant positive correlations between forecasts and observed outcomes. While the forecasts can help to provide a preliminary assessment of preprints at a faster pace than traditional peer-review, it remains to be investigated if such an assessment is suited to identify methodological problems in preprints.

## 1. Introduction

The quick rise of the COVID-19 pandemic was followed by an unprecedented explosion in COVID-19-related research [1–5]. The largest increase in the volume of academic papers from the previous year was in 2020 [6], and at the time of writing *PubMed* contained nearly 170 000 COVID-19-related publications in its database. The dynamics of the pandemic necessitated research findings to be disseminated quickly to other researchers as well as policy and decision makers. Preprint servers helped to accelerate this process by making data and research findings accessible without delays associated with traditional publications including peer-review [7–9]. The fast turnaround was credited with helping to mitigate the potential impacts of the pandemic, including saving lives [1,10]. It has been estimated that once the pandemic became widespread in early 2020, 40–50% of all COVID-19-related publications were submitted to preprint servers before entering the traditional academic publishing route [2,3]. COVID-19 dominated preprint servers, with over half of all preprints on *medRxiv* being COVID-19-related in every month between March 2020 and August 2021 [6]. Preprints typically differ from published manuscripts in that they are shorter and contain fewer references [3]. In addition, the type of research appearing in preprints differs from traditional publications, with randomized controlled trials, systematic reviews, and observational findings appearing more often in preprints, and traditional publications including more case reports and letters [2].

The benefit of a faster dissemination of results comes potentially at the cost of a lower reliability of the released findings [1,4,7,11–13]. While benefits and risks of preprints are well documented, a consensus on whether the benefits outweigh the risks has not been reached [14–19]. Many preprints are not at ‘publication quality’ and have flaws in data or methods [1]. This raises concerns when findings from preprints are shared in traditional and social media, even with the proviso by preprint servers that the manuscript has not undergone peer-review [20,21]. Due to their speed, preprints rather than peer-reviewed publications can be the focus of discourse [4]. While people of academic and non-academic backgrounds interact with preprints on social media such as *Twitter*, the difference between peer-reviewed and non-peer-reviewed research may not be understood and flawed studies may be disseminated through media [12,20–22]. This problem can be exacerbated by media searching for ‘scoops’ and therefore focusing on exciting and new, but potentially unreliable, findings in preprints [8].

It has been estimated that only 5–14% of COVID-19-related preprints will be published [3,23,24] and preprints which are eventually published often undergo significant changes, in part due to the peer-review process [13]. While remaining unpublished for a prolonged time can be an indication of the quality of a preprint, some preprints have been so erroneous in their claims that they have been retracted. The latter includes one infamous preprint which reported that the COVID-19 virus contained HIV ‘insertions’ [1,4]. It should, however, be noted that even peer-reviewed published findings cannot be assumed to always be correct or without error [25,26]. Moreover, publication in a high-impact journal or a high citation count are not reliable indicators for the methodological quality of a study [27,28].

In the COVID-19 preprint forecasting study, we investigate if forecasting can help to fill the gap from preprints lacking peer-review. It is currently unknown if crowd-based forecasting allows us to identify which preprints are useful and robust and which preprints have flaws which will prevent publication in an academic journal. We asked forecasters to predict publication outcomes and future citations of 400 preprints uploaded on preprint servers between 1 January 2020 and 31 August 2020.

The selection of preprints was based on *Altmetric* scores. *Altmetric* scores are an alternative to traditional impact measures such as citations and are calculated based on metrics such as (but not limited to) mentions on social media, mainstream media coverage, discussion on research blogs and citations on Wikipedia [29]. We split our sample into 10 bins by time and selected the top 40 preprints in each bin as ranked by *Altmetric*. This sampling method was designed so we could test the most widely disseminated preprints across an extended period, which can be expected to include the most relevant findings. We conducted incentivized surveys in November 2020, asking forecasters to predict the probabilities of three possible futures of each preprint: (i) remaining unpublished within 1 year of dissemination, (ii) being published in a medium- or low-impact journal or (iii) being published in a high-impact journal. We defined medium or low impact as a journal impact factor (JIF) below 10 and high impact as a JIF of at least 10. In addition, we asked forecasters to predict how a preprint will rank in terms of citations received after 1 year, relative to the other preprints, with a rank of 0 assigned to the least cited preprint and a rank of 100 to the most cited preprint. We also elicited more subjective assessments such as ‘usefulness’ and replicability. Previous research has shown that forecasters can predict characteristics of papers including their replicability [30–32]. While we will not ‘resolve’ the subjective assessments of preprints, these forecasts serve as a measure of expected usefulness and replicability.

The nature of preprints provides an opportunity for a crowd to provide informal assessments prior or in parallel to more formal assessment through a few peer-reviewers and an editor [8]. Our study seeks to understand the extent to which the future publication and citation outcomes of preprints can be forecasted. Our approach can help to inform future policy around the use of preprints. In addition, our forecasts can yield an interim measure for the potential impact of a manuscript.

## 2. Methods

Our sample contains 400 COVID-19 preprints from the *medRxiv* and *bioRxiv COVID-19 SARS-CoV-2* collection (connect.biorxiv.org/relate/content/181) uploaded before the end of August 2020. Of these 400 preprints, 92 were found on *bioRxiv* and 308 were found on *medRxiv*. To focus on preprints that have been recognized by media and social media to be of high relevance, we divided the roughly 8000 preprints in the entire collection at the time of sampling into ten bins by time and selected the top 40 within each bin as ranked by the *Altmetric* score. This stratified sampling strategy results in an even distribution of preprints across time and avoids the sample being dominated by earlier preprints which had more time to gain a higher *Altmetric* score. Any preprints that had been indicated by *medRxiv* or *bioRxiv* to already be published were excluded from the sampling process. Because of the delay between sampling preprints and conducting the surveys and because publication indicators on preprint servers are not comprehensive, 153 preprints had already been published by the end of the survey period (10 November 2020), leaving 247 preprints that were not published by the end of the forecasting period. For hypothesis tests that were not related to forecasting, we used the full set of 400 preprints. Our primary analysis of forecasting performance will focus on these 247 preprints because forecasters could have been aware of the publication outcome for the remaining 153 preprints. However, we will provide tests on the full dataset as well.

Participants were recruited predominantly from forecasters in the Systematizing Confidence in Open Research and Evidence (SCORE) [32] project, which focused on forecasting the replicability of scientific claims in the social and behavioural sciences. Additionally, participants were recruited via social media, primarily *Twitter* and *Reddit*. We had 49 participants in total. The participants are typically but not necessarily from academia or have academic backgrounds, and since the SCORE project focused on claims from the social and behavioural sciences, most participants had experience in these fields rather than in biomedical research. Of the 49 participants, 31 stated in an entry questionnaire to have at least one scientific publication, and 13 stated to have at least one publication in the biomedical sciences. In terms of career stage, 47% were graduate students, 27% were early career researchers (for instance postdoctoral researchers or assistant professors) and 17% were mid-career researchers (e.g. senior research fellow or associate professors). Senior researchers make up only 3% of the participants. The majority of participants (61%) worked or studied in academia when the survey was taken, with a further 24% working in industry.

Participants were assigned an initial random ‘batch’ of 10 preprints; once the initial batch was completed, participants had the option to complete additional batches. New batches were assigned to the participants after each batch they completed. Participants only learned about the preprints in a batch after it had been assigned to them. Participants could not select into assessing specific preprints and could after each batch solely choose to continue or discontinue with forecasting.

The median number of preprints forecasted by participants is 30 (*M =* 93, s.d. *=* 133). Conversely each preprint has a median number of forecasts of 11 (*M* = 11.44, s.d. = 1.98, Min = 6, Max = 15). The surveys were open from 28 October 2020, through 10 November 2020. Participants had no access to other participants' forecasts. The abstract of the preprints was provided within the survey along with links to the full version of the online preprint. The surveys included four forecasting questions (with answers or prompts):

— **(Q1)** Will this preprint be published in a peer-reviewed scientific journal within a year of first preprint posting? Provide a % probability between 0 and 100 for each option. The values must sum to 100: (option 1) No, not published, (option 2) Yes, in a journal with impact factor below 10, (option 3) Yes, in a journal with impact factor of at least 10.
— **(Q2)** Rank this preprint's 1-year Google citations count relative to other preprints in this study. Select (using slider) a relative rank between 0 for least cited and 100 for most cited.
— **(Q3)** What is the % probability that the findings presented in the preprint agree with the majority of results from similar future studies? Select (using slider) a value between 0 (impossible) and 100 (certain).
— **(Q4)** Are the results presented in the preprint helpful to mitigate the impact of the COVID pandemic? Select (using slider) a value between 0 (no) and 100 (yes).

Because experiences from previous projects suggest that participants often provide ‘conflated’ responses when asked about different aspects of a publication, we asked Q3 and Q4 in random order. Incentives for the surveys were provided through surrogate scoring [33]. This method does not require access to a ‘ground truth’ outcome to incentivize truthful reporting and is thus well suited to elicit a broader range of judgements. It is also well suited to generate accuracy estimates for paying prizes without delay if resolution is not available immediately, such as in this case. The core idea of the surrogate scoring rule (SSR) is to first identify a surrogate outcome using the collected judgement (the mean). Then, we develop a statistical estimation procedure to uncover the bias in this surrogate and noisy outcome. This knowledge of the bias helps us define unbiased estimates of the true scores as if we had access to the ground truth outcome, using only the surrogate outcome [33]. Prizes for the surveys were given for the best forecasters in each batch. With 40 batches of 10 questions, we paid USD 90 per batch, paid as $30, $25, $20 and $15 to the first, second, third and fourth place for that batch, as determined by the surrogate scoring method. Participants could participate and win prizes in more than one batch.

The questions Q1 and Q2 have been resolved 1 year after the preprint was uploaded to a preprint server. Publication outcomes were resolved manually using *Google Scholar* searches for any published version of a particular preprint. Resolutions allowed for some differences in preprints and publications such as updated sample sizes or edited text. In cases where published articles were made available online before the official journal edition publication date, the earliest date was used as the publication date. The JIF in 2020 was used for resolving published preprints. The citation counts were also resolved manually using the total number of citations recorded by *Google Scholar*, summing-up citation counts of multiple instances of the paper (including preprints and published version) being indexed by *Google Scholar*. Where preprints were not resolved after 1 year (which applies to 86 preprints), citation counts were backdated using *Google Scholar*'s citations by date function. Q3 and Q4 are not resolved but serve as a gauge of the expectations of the forecasters of the robustness and helpfulness (regarding the mitigation of the pandemic) of the preprints. We also conducted a prediction market to forecast above outcomes but decided not to use the corresponding data because the starting prices were set incorrectly, and prices on the dashboard did not update correctly. The combination of these technical issues coupled with a low participation of only around 30 traders led us to not analyse the prediction markets, although the unused data will be made available. This decision was made before any data were analysed.

The experimental design and statistical analyses were preregistered on the *Open Science Framework* (OSF; osf.io/tb9ms/) after the surveys were completed, towards the end of the data collection for the resolutions. We did not use the surveys or incomplete outcome data for any preliminary analyses. Any deviations from the preregistered analyses or any added non-preregistered tests will be mentioned in the text below. For statistical tests, we interpret the threshold of *p* < 0.005 as identifying statistical significance, and the threshold of *p* < 0.05 as identifying suggestive evidence [34]. The statistical analysis was completed in ‘*R*’, using the ‘*miceadds*’ package for applying clustered standard errors [35,36].

## 3. Results

Of the 400 preprints in our sample, 281 (70%) were published in an academic journal within 1 year. Of the 247 preprints that were not published until the end of the forecasting period, 128 (52%) were published within 1 year. One hundred and nineteen preprints (30% of the full sample) were not published within a year after the upload to the preprint server. The median time between the release date on a preprint server and becoming published is 143 days (*M* *=* 151, s.d. *=* 81, Min *=* 14, Max *=* 362). Note that more preprints in our sample may have been published or will be published outside our 1-year cut-off. One hundred and twenty-seven of the 281 (45%) published papers were published in a high-impact journal (JIF of at least 10). Papers were published in 135 different journals with the most common being *Nature* (22 papers), *Science* (16 papers) and *Nature Communications* (14 papers).

We also collected citation counts for each of the 400 preprints, combining *Google Scholar* citation counts for all versions including preprints and published versions (where relevant). The median number of citations after one year (from the release date on a preprint server) is 54 (*M* *=* 162, s.d. *=* 308, Min *=* 14, Max *=* 3,286). Fifty-five papers were cited less than 10 times, 200 papers were cited between 10 and 100 times, 138 papers were cited between 100 and 1000 times, and 8 papers were cited more than 1000 times. We transformed citation counts into relative ranks between 0 and 100, where a paper with rank 0 has the lowest citation count and a paper with rank 100 has the highest citation count. These ranks were used in our statistical analyses (instead of the actual citation counts) with the advantage of avoiding inferential problems that may arise due to the strongly right-skewed distribution of citation count data.

Publication status (i.e. whether or not the paper has been published) is statistically significantly correlated with the paper's citation rank (Pearson correlation, *r* = 0.41, *t*_398_ = 8.91, *p* < 0.0001), with non-published preprints having an average citation rank of 32 compared with published preprints' average rank of 58, a statistically significant difference (Welch two-sample *t*-test, not preregistered, *t*_263.46_ = 9.57, *p* < 0.0001). For the preprints which have been published, citation ranks are statistically significantly correlated with the impact factor category (*r* = 0.50, *t*_273_ = 9.60, *p* < 0.0001) where impact factor category refers to a binary variable indicating publication in a high-impact or medium- to low-impact journal. Preprints published in journals with impact factors of at least 10 have an average citation rank of 73 compared with an average citation rank of 45 for preprints published in journals with a JIF below 10 (Welch two-sample *t*-test, not preregistered, *t*_271.41_ = 9.65, *p* < 0.0001). Preprints published in low- to medium-impact journals (JIF < 10) were cited statistically significantly more often than non-published preprints (Welch two-sample *t*-test, not preregistered, *t*_259.02_ = –4.44, *p* < 0.0001).

**Figure 1.**
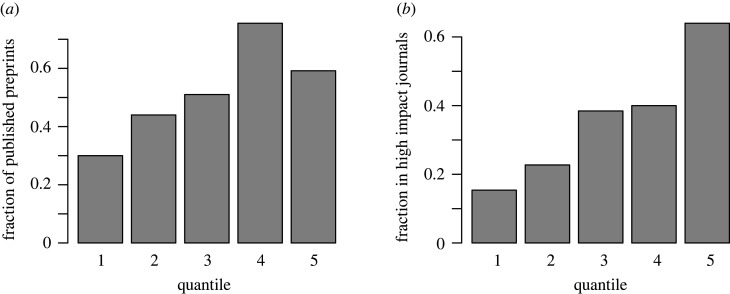
Forecasts of publication outcomes: (*a*) shows the fraction of published preprints depending on the mean forecasted probability. Of the preprints with a lowest forecasted probability of being published within 1 year (lowest quantile), about 30% were published. For the highest quantile, this fraction is about 60%. Publication outcomes are correlated with mean forecasts (*r* = 0.23, *t*_245_ = 3.69, *p* < 0.001). Preprints for which the publication status was known during the forecasting period were excluded from this figure; (*b*) shows the fraction of preprints published in high-impact journals depending on the corresponding forecast. Of the preprints with the lowest forecasted probability (lowest quantile) of being published in a high-impact journal, only about 15% were published in a high-impact journal. For the highest quantile, this fraction is over 60%. High-impact publication is correlated with predicted high-impact publication (*r* = 0.38, *t*_122_ = 4.58, *p* < 0.001). Unpublished preprints and preprints for which the publication status was known during the forecasting period were excluded from this figure.

Using the subset of preprints that were not published by the end of the forecasting period, the mean forecasts for publication status (combining forecasts for publication in any journal in this instance) are statistically significantly positively correlated with the binary publication outcome (0 = not published, 1 = published; *r* = 0.23, *t*_245_ = 3.69, *p* = 0.0003; figure 1). When including already published preprints (i.e. the full 400 preprint sample), the correlation increases to 0.35 (*r* = 0.35, *t*_398_ = 7.46, *p* < 0.0001), potentially indicating that the forecasters were aware of at least some of the known publication outcomes. We also regressed individual responses against publication outcomes in a linear probability model with s.e. clustered at the forecaster level. The clustering of s.e. at the forecaster level is to avoid biased s.e. as the set of predictions by each forecaster is unlikely to be independently and identically distributed (i.e. within cluster correlation). This model provides suggestive evidence for a positive association between individual forecasts for publication in any journal and the binary publication outcome (*β* = 0.16, *t*_47_ = 2.53, *p* = 0.012). When assessing binary accuracy, where we interpret an average survey forecast of less than 0.5 as a prediction of a preprint not being published, and an average survey forecast of 0.5 or above as a prediction of a preprint being published, forecasters had an accuracy of 59% in the sample of preprints that were not published by the end of the forecasting period. There is suggestive evidence that this accuracy rate is better than random chance (binomial test, not preregistered, *p =* 0.0074).

Forecasters statistically significantly overestimated the overall publication rate when comparing binary publication outcomes and average forecasts of probability of publication (for preprints with unknown publication outcomes, *n* = 247): forecasters expected 65% of preprints to be published compared to the reality of 52% (paired *t*-test, *t*_246_ = 4.07, *p* < 0.0001). Considering the full sample of preprints, the average forecasts of 68% match the observed publication rate of 70% much better, though this comparison comes with the caveat that some participants might have already known the correct publication outcome.

We also tested if forecasters could predict the impact factor of the journal the preprint will be published in. Focusing on the subset of preprints not published before the end of the forecasting period, we found a statistically significant correlation between the mean survey predictions for being published in a high-impact journal (given that it is published) and the binary outcome (0 = published in low-medium impact journal and 1 = published in a high-impact journal, *r* = 0.38, *t*_122_ = 4.5773, *p* < 0.0001; figure 1). In addition, a linear probability model was used on the individual level responses with clustered s.e. at the forecaster level. This model shows a positive association between forecasts and outcomes (*β* = 0.27, *t*_47_ = 4.33, *p* < 0.0001). There is suggestive evidence that forecasters tended to underestimate the share of papers which were published in high-impact journals (forecast average of 27% versus observed 36%, paired *t*-test, *t*_123_ = –2.22, *p* = 0.029).

**Figure 2.**
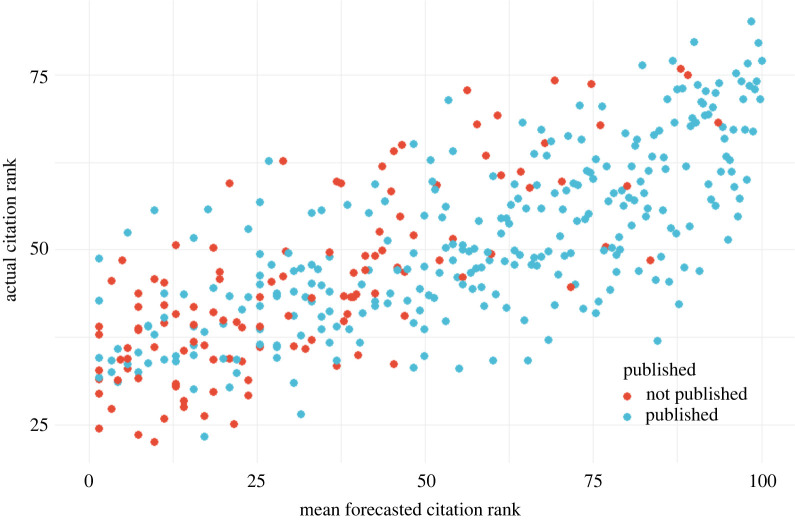
Actual citation ranks versus forecasted citation ranks. This plot demonstrates the relationship between mean forecasted citation ranks and actual citation ranks. The colour of the markers indicates the publication status (irrespective of the impact factor of the journal), with blue markers indicating published preprints and red markers indicating preprints not published within 1 year. While the mean forecasts are highly correlated with realized citation ranks (*r* = 0.75, *t*_398_ = 22.36, *p* < 0.001), the aggregated forecasts are not extreme enough with few preprints forecasted to be ranked below 25 or above 75.

Forecasters can predict which preprints will have few or many citation counts after 1 year. Using the full sample, citation ranks (from 0 to 100) are statistically significantly correlated with the mean forecasts for citation ranks (*r* = 0.75, *t*_398_ = 22.358, *p* < 0.0001). The strength of this relation is illustrated in figure 2. This relationship remains similarly strong when excluding preprints with known publication outcomes (un-preregistered *r* = 0.69, *t*_245_ = 14.803, *p* < 0.0001).

As the preprints in our sample were released at different times, we tested if papers which were released early, and therefore had more time to get indications of citations, had a systematically lower absolute error. We correlated days between upload to preprint server and the end of the survey period (10 of November 2020) and absolute citation rank error in an un-preregistered test. We found no evidence that being released earlier correlates with more accurate forecasts (*r* = –0.10, *t*_398_ = –1.9214, *p* = 0.0554). In addition, we also correlated days between upload to preprint server and the end of the survey period and absolute forecasting error for publication and JIF outcomes. Forecasts of publication outcomes or JIF are not correlated with how early the preprint was released (*r* = –0.0388, *t*_398_ = –0. 77536, *p* = 0.4386; *r* = –0.0798, *t*_122_ = –0. 8841, *p* = 0.3784).

The survey responses were incentivized using SSR which provides estimates of a forecaster's AUC or Brier score. AUC (Area under the ROC Curve) and the Brier score are both measures of accuracy of a predictor of a binary outcome. The AUC of a predictor is equal to the probability that the predictor will rank a random positive outcome higher than a random negative outcome. The Brier score is equivalent to the mean squared error [37]. We tested the accuracy of SSR in identifying the most accurate forecasters. Using SSR, we estimated the rank of users by AUC and Brier score in each batch (a batch is made up of 10 preprints) and compared the estimated ranks with actuals. We found that estimated ranks of batchwise AUC and Brier score are correlated with actual ranks (AUC: *r* = 0.16, *t*_471_ = 3.55, *p* = 0.0004; Brier Score: *r* = 0.17, *t*_471_ = 3.66, *p* = 0.0003). We also correlated overall users' SSR estimated AUC and Brier Scores with actual AUC and Brier Scores. We found no statistically significant correlation between overall SSR estimates and actual AUC (*r* = –0.10, *t*_30_ = –0.54, *p* = 0.5954) or Brier Score (*r* = 0.29, *t*_30_ = 1.67, *p* = 0.1057). SSR can also be used to aggregate forecasts by taking an average of only the forecasters with highest estimated AUC, with the aim to remove uninformative forecasts. We found evidence that squared error of SSR aggregated citation rank is higher than the squared error of the mean aggregated citation rank (paired *t*-test, *t*_399_ = –10.63, *p* < 0.0001).

In addition to the resolvable questions of publication and citation outcomes, we also asked forecasters to provide subjective judgements on the replicability and usefulness (with respect to the mitigation of the pandemic) of the preprints. While not being able to determine the accuracy of these forecasts, they serve as a gauge of the expectations of how the findings in our sample sit in the wider body of COVID-19 literature. We used the full sample of 400 preprints for all tests here, except for tests that include forecasts of publication and journal outcomes for which we used the subset of preprints where publication outcome was unknown at time of the surveys (*n* = 247). We found that average responses to Q3 (What is the % probability that the findings presented in the preprint agree with the majority of results from similar future studies?) correlate positively with forecasts for publication (*r =* 0.64, *t*_245_ = 13.12, *p* < 0.0001), forecasted citation rank (*r* = 0.52, *t*_398_ = 12.02, *p* < 0.0001) and forecasted usefulness (*r =* 0.62, *t*_398_ = 15.74, *p* < 0.0001). In terms of outcomes, forecasts for agreement (Q3) are also positively correlated with being published (*r =* 0.36, *t*_398_ = 7.72, *p* < 0.0001), being published in a high-impact journal (*r =* 0.24, *t*_273_ = 4.15, *p* < 0.0001), and the citation rank (*r =* 0.43, *t*_398_ = 9.39, *p* < 0.0001). The assessed usefulness of preprints (as measured by Q4) is also correlated with forecasts of being published (*r =* 0.70, *t*_245_ = 15.34, *p* < 0.0001) and forecasts of being published in a high-impact journal (*r =* 0.65, *t*_245_ = 13.28, *p* < 0.0001). Moreover, the forecasts for usefulness are positively correlated with observed publication in a high-impact journal (*r =* 0.33, *t*_273_ = 5.87, *p* < 0.0001) and with actual citation ranks (*r =* 0.50, *t*_398_ = 11.46, *p* < 0.0001).

As participants may provide conflated answers for Q3 and Q4, we randomized the order of these questions with half of the participants being asked Q3 first; the other half Q4 first. To test for order effects, we compared responses to Q3 and Q4 between those who answered Q3 or Q4 first using unpaired *t*-tests. We found that responses are significantly lower when Q3 is asked first as opposed to asked second (Welch two-sample *t*-test, difference = –0.07, *t*_545.26_ = –7.10, *p* < 0.001). No statistically significant order effects were found for Q4 (Welch two-sample *t*-test, difference = –0.01, *t*_558.70_ = –0.90, *p* = 0.370). All correlations including Q3 were split by whether it was asked first or second can be found in table 1.

**Table 1.** Pairwise Pearson correlation coefficients of survey questions and outcomes. The order of questions 3 and 4 was randomized so that some participants always saw question 3 first and some always saw question 4 first. We found evidence for order effects for question 3 and so included all answers for question 3 (labelled ‘Q3—agreement with other papers' in the table) and split by whether it was asked first or second.

	Q1: not published	Q1: published (IF < 10)	Q1: published (IF > 10)	combined published forecast (any IF)	Q2: cite Rank	Q3: agreement with other papers	Q3: agreement with other papers (asked first)	Q3: agreement with other papers (asked second)	Q4: helpful	publication outcome	published (IF > 10)
Q1: published (IF < 10)	–0.71 (*p* < 0.0001)										
Q1: published (IF > 10)	–0.73 (*p* < 0.0001)	0.05 (*p* = 0.4806)									
combined published forecast (any IF)	–1.00 (*p* < 0.0001)	0.71 (*p* < 0.0001)	0.73 (*p* < 0.0001)								
Q2: cite rank	–0.78 (*p* < 0.0001)	0.44 (*p* < 0.0001)	0.68 (*p* < 0.0001)	0.78 (*p* < 0.0001)							
Q3: agreement with other papers	–0.64 (*p* < 0.0001)	0.50 (*p* < 0.0001)	0.43 (*p* < 0.0001)	0.64 (*p* < 0.0001)	0.52 (*p* < 0.0001)						
Q3: agreement with other papers (asked first)	–0.53 (*p* < 0.0001)	0.38 (*p* < 0.0001)	0.38 (*p* < 0.0001)	0.53 (*p* < 0.0001)	0.50 (*p* < 0.0001)	0.90 (*p* < 0.0001)					
Q3: agreement with other papers (asked second)	–0.56 (*p* < 0.0001)	0.45 (*p* < 0.0001)	0.35 (*p* < 0.0001)	0.56 (*p* < 0.0001)	0.35 (*p* < 0.0001)	0.76 (*p* < 0.0001)	0.46 (*p* < 0.0001)				
Q4: helpful	–0.70 (*p* < 0.0001)	0.36 (*p* < 0.0001)	0.65 (*p* < 0.0001)	0.70 (*p* < 0.0001)	0.69 (*p* < 0.0001)	0.62 (*p* < 0.0001)	0.56 (*p* < 0.0001)	0.48 (*p* < 0.0001)			
publication outcome	–0.23 (*p* = 0.0003)	0.21 (*p* = 0.0010)	0.13 (*p* = 0.0489)	0.23 (*p* < 0.0001)	0.25 (*p* < 0.0001)	0.36 (*p* < 0.0001)	0.32 (*p* < 0.0001)	0.33 (*p* < 0.0001)	0.23 (*p* < 0.0001)		
published (IF > 10)	–0.23 (*p* = 0.0003)	0.02 (*p* = 0.8127)	0.31 (*p* < 0.0001)	0.23 (*p* < 0.0001)	0.41 (*p* < 0.0001)	0.30 (*p* < 0.0001)	0.29 (*p* < 0.0001)	0.23 (*p* < 0.0001)	0.33 (*p* < 0.0001)	0.45 (*p* < 0.0001)	
actual cite rank	–0.54 (*p* < 0.0001)	0.31 (*p* < 0.0001)	0.47 (*p* < 0.0001)	0.54 (*p* < 0.0001)	0.75 (*p* < 0.0001)	0.43 (*p* < 0.0001)	0.43 (*p* < 0.0001)	0.27 (*p* < 0.0001)	0.50 (*p* < 0.0001)	0.41 (*p* < 0.0001)	0.54 (*p* < 0.0001)

## 4. Discussion

The forecasting of scientific outcomes can help to improve science [30,31,38–41]. In previous projects, it has been shown that forecasters can predict replicability of published studies [30,40], and effect sizes [42,43]. While earlier studies used forecasting to predict the rating of publications in review exercises such as the *Research Excellence Framework* [44], this is the first project to forecast outcomes pertinent to specific preprints. The use and function of preprints has changed over the course of the global COVID-19 pandemic—providing potentially lifesaving findings and data to policy makers [1,10]. The undeniable benefits of preprints do come at a cost—the lack of peer-review can result in some preprints lacking credibility. We sought to understand the extent to which publication and citation outcomes for preprints can be forecasted. We elicited through incentivized surveys; forecasted the probability of publication in a peer-reviewed journal (with three outcomes including impact factor of journal), forecasted relative citation rank and two non-verifiable characteristics of the preprint—the agreement with other results (i.e. replicability) and the usefulness of mitigating the impact of the pandemic.

Our results show that forecasters can predict if preprints will be published within 1 year, despite overestimating the share of preprints in our sample which will be published. In total, we found 70% of preprints were published within a year—a number that can be expected to increase as some preprints will take longer than a year to publish. This rate of publication is in line with other research which has shown that around two-thirds of preprints are eventually published [45–47] and is larger than the estimate that only 5–14% of COVID-19-related preprints will be published [3,23,24]. Note that the *Altmetric*-based sampling we used in our study likely translates into a higher publication rate for our sample compared to more comprehensive COVID-19 preprint samples.

Forecasters were also able to predict the impact of journals in which the preprints will be published, despite underestimating the number of the papers which are published in high-impact journals. The observed fraction of 45% of the published preprints appearing in high-impact journals is larger than our expectation and is likely substantially influenced by topic and sampling methods. Similarly, forecasts of relative citation ranks are highly correlated with actual relative citation ranks. We also found that subjective assessments of replicability and usefulness are correlated with publication outcomes and citation ranks. Our forecasters were more accurate at predicting citation rank than publication outcomes. We excluded a number of preprints from our accuracy analysis due to them being already published by the time of the forecasting survey. Preprints which are published quickly may be more obviously ‘publishable’ and therefore are easier to forecast. Preprints which are delayed in being published and therefore are in our sample may be more difficult to forecast. In addition, there is a delay between the prints in our sample being uploaded to a preprint server and the forecasting survey. This means that participants had some information on citations when forecasting. We found that having more information on citations (from preprints being uploaded earlier from the surveys) is not statistically significantly correlated with more accurate predictions of final citation ranks. Finally, it may be that citations are a more fine-grained and continuous outcome than publication and therefore easier to forecast. Further study with forecasting happening shortly after preprints are released may be used to test these assumptions.

Our findings are promising in that they indicate that forecasting can be used to help fill the gap left by the lack of peer-review on preprints. While assessments such as the ones used in our study can be driven by factors that are unrelated to the quality of the methodology behind a preprint, it would be in line with previous findings from forecasting projects associated with large-scale replication studies [30,40] that the perceived credibility of a study informs assessments. Further research is required to identify which features of a preprint are used by forecasters for their assessment and to establish if the findings presented here apply to other topics and research fields.

## Data Availability

Data and code are available from the Dryad Digital Repository: https://doi.org/10.5061/dryad.rfj6q57d0 [48].
